# Molecularly Imprinted Polymer Advanced Hydrogels as Tools for Gastrointestinal Diagnostics

**DOI:** 10.3390/gels11040269

**Published:** 2025-04-04

**Authors:** Tatjana Ivaskiene, Greta Kaspute, Arunas Ramanavicius, Urte Prentice

**Affiliations:** 1 State Research Institute Centre for Innovative Medicine, LT-08410 Vilnius, Lithuania; tatjana.ivaskiene@imcentras.lt (T.I.); greta.kaspute@ftmc.lt (G.K.); 2 Department of Nanotechnology, State Research Institute Center for Physical Sciences and Technology (FTMC), LT-10257 Vilnius, Lithuania; 3 Department of Physical Chemistry, Faculty of Chemistry and Geosciences, Institute of Chemistry, Vilnius University, LT-03225 Vilnius, Lithuania

**Keywords:** molecularly imprinted polymers, synthetic receptors, biosensors, gastrointestinal disorders, biomedical diagnostics

## Abstract

Gastroenterology faces significant challenges due to the global burden of gastrointestinal (GI) diseases, driven by socio-economic disparities and their wide-ranging impact on health and healthcare systems. Advances in molecularly imprinted polymers (MIPs) offer promising opportunities for developing non-invasive, cost-effective diagnostic tools that enhance the accuracy and accessibility of GI disease detection. This research explores the potential of MIP-based sensors in revolutionizing gastrointestinal diagnostics and improving early detection and disease management. Biomarkers are vital in diagnosing, monitoring, and personalizing disease treatment, particularly in gastroenterology, where advancements like MIPs offer highly selective and non-invasive diagnostic solutions. MIPs mimic natural recognition mechanisms, providing stability and sensitivity even in complex biological environments, making them ideal for early disease detection and real-time monitoring. Their integration with advanced technologies, including conducting polymers, enhances their functionality, enabling rapid, point-of-care diagnostics for gastrointestinal disorders. Despite regulatory approval and scalability challenges, ongoing innovations promise to revolutionize diagnostics and improve patient outcomes through precise approaches.

## 1. Introduction

Gastroenterology has emerged as a critical field in present-day medicine due to the increasing ubiquity of gastrointestinal (GI) and digestive diseases and their profound impact on global health [1]. Digestive diseases, driven by enteric infections in lower-income regions and colorectal cancer in higher-income areas, account for a significant global disease burden, with prevalence and impact closely tied to socio-economic conditions [1]. Gastrointestinal diseases like peptic ulcer disease [2], gastritis [3], duodenitis, gastroesophageal reflux disease [4], and celiac disease [5] pose global healthcare challenges, reflecting disparities in socio-economic development and healthcare access [6] in both low-income and high-income regions. GI diseases are significant because they cause widespread suffering, can lead to fatal outcomes, and place a heavy burden on healthcare systems through frequent hospitalizations and high costs. Analyzing hospital discharge data [7,8], previous studies determined that GI conditions were common as primary diagnoses and frequently arose as secondary issues, often complicating patient care [7]. The results of GI disease research from studies in both the United States and Europe [9] show a significant burden, with millions of healthcare encounters, hundreds of thousands of deaths [10], and billions in annual costs, as these conditions are highly prevalent worldwide [10].

Understanding these patterns highlights the importance of early detection strategies to reduce their impact on patients and healthcare systems alike. Accurate diagnostic tools are crucial for GI disease as their varied clinical presentations often lead to misdiagnosis or delayed diagnosis, and current methods, including serological tests (for celiac disease and others), suffer from inconsistencies and limited global accessibility [5]. Currently, the most appropriate diagnostics in gastroenterology are colonoscopy, gastroscopy, and/or biopsy [11,12,13]. These invasive diagnostics are uncomfortable and often require sedation [14], which can discourage patients from undergoing examinations. Therefore, new technology and methods for using biomarkers are being developed, which will help in the future to more easily and, in some cases, more accurately determine conditions or stages of GI disorders. However, many challenges arise in this area because the biomarkers used in GI are characterized by deficiencies in specificity and sensitivity, so current biomarkers of GI diseases (e.g., fecal calprotectin, C-reactive protein) may not reliably distinguish between conditions or stages of the disease [15,16,17]. New biomarkers and new technologies in the field of sensors are very promising [18,19,20,21,22,23].

Recent progress in analytical chemistry enables the design of novel analytical systems based on synthetic receptors [24] using molecularly imprinted polymers (MIPs) [25,26,27]. The importance of advancing MIP-based diagnostic tools is becoming increasingly evident [28]. This issue became extremely important during the wake of the COVID-19 pandemic, which highlighted the critical role of rapid tests and sensors [29,30], including sensors based on MIPs due to the SARS-CoV-2 virus spike [31] and nucleocapsid [32] proteins. Improved diagnostics are essential for the early detection of diseases, as many conditions, such as colorectal cancer [33] and inflammatory bowel diseases (IBD) [34,35], begin with non-specific or asymptomatic presentations, leading to delayed diagnoses and poorer outcomes [36]. Considering these developments in the determination of protein-based biomarkers, similar advanced sensors are also needed to differentiate between overlapping symptoms of common gastrointestinal disorders while minimizing the discomfort and invasiveness associated with traditional methods like colonoscopies. Non-invasive, cost-effective technologies, including molecular sensors and biomarkers, not only enhance accessibility but also align with the growing demand for personalized medicine by enabling precise, tailored treatments based on individual profiles. Furthermore, emerging challenges, from antibiotic-resistant infections to rare GI cancers [37], underscore the need for sophisticated approaches.

This integrated summary presents the potential of applying MIP sensors in gastrointestinal diagnostics while also exploring future perspectives in the field.

## 2. Methods

**Figure 1:**
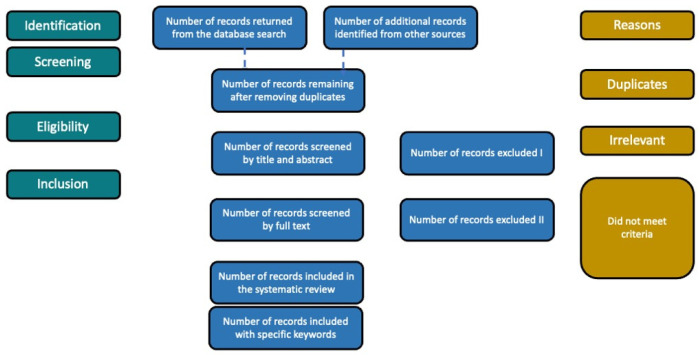
Adapted PRISMA-type [38] literature search strategy.

To address the research question of how molecularly imprinted polymer (MIP) advanced hydrogels serve as tools for gastrointestinal diagnostics, we conducted a systematic literature search across three major scientific databases: ScienceDirect (Scopus), Web of Science, and PubMed. The search was performed in 2025 and included English-language studies without restrictions on publication year to ensure a comprehensive review (Figure 1). The queries focused on key terms such as “Molecularly Imprinted Polymers” and “Gastrointestinal Diagnostics,” as well as broader searches using “MIP” and “in vivo” to capture a wide scope of relevant research. The initial results yielded a large number of studies, with ScienceDirect returning 1266 papers for MIPs in gastrointestinal diagnostics and over 35,000 for MIPs in general, while Web of Science and PubMed produced fewer but highly specific results.

To refine the selection, duplicate articles were removed by identifying redundant DOI entries across platforms. Studies that did not specifically investigate or fit MIPs in gastrointestinal diagnostics were excluded, alongside papers which only briefly mentioned the topic without providing substantial experimental or analytical depth. Preference was given to research that included detailed methodologies, experimental validation, and/or clinical applications. Additionally, studies from the authors’ working group were incorporated due to their direct relevance and alignment with the research objectives. To maintain focus, if a paper presented clear references to key ideas, no further sources were added merely to increase volume.

By applying these criteria, the final selection of articles ensured a strong foundation for understanding the role of molecularly imprinted polymer hydrogels in gastrointestinal diagnostics. This process streamlined the dataset and highlighted high-value contributions in the field, ensuring that the included research offered meaningful insights rather than redundant or marginally relevant findings.

## 3. Current GI Diagnostics and Possibilities of Novel Tools’ Integration

Gastrointestinal diseases can be localized to the GI tract, such as reflux esophagitis and peptic ulcers, or be associated with systemic disorders like IBD. They may even cause systemic issues through primary GI pathologies, such as vitamin deficiencies from malabsorption [39]. The nature of the disease varies by the specialized function of the affected GI region, leading to different causes and manifestations [39]. For example, irritable bowel syndrome (IBS) is a GI disorder marked by changes in bowel habits accompanied by abdominal discomfort or pain without identifiable structural or biochemical abnormalities. Its pathogenesis involves various factors, including altered GI motility, visceral hypersensitivity, post-infectious responses, brain–gut interactions, changes in gut microbiota, bacterial overgrowth, food sensitivities, carbohydrate malabsorption, and intestinal inflammation [40,41,42]. The variety of possible causes of GI disorders leads to the need for early diagnostic or non-invasive tools to detect the issue. Emerging diagnostic technologies, like liquid biopsy and advanced molecular imaging, offer non-invasive ways to detect GI disorders early, enabling timely interventions before symptoms appear. Integrating genetic and omics data enhances predictive models, helping identify high-risk individuals and inform personalized prevention strategies. Additionally, treatment approaches are evolving, with precision medicine guiding the development of targeted therapies, including immunotherapies, gene therapies, and microbiome-based interventions, which could revolutionize the management of conditions such as inflammatory bowel disease and diabetes [41,43]. Another opportunity is to apply electrochemical methods to develop biosensors that can be applied in GI disease diagnostics.

**Figure 2:**
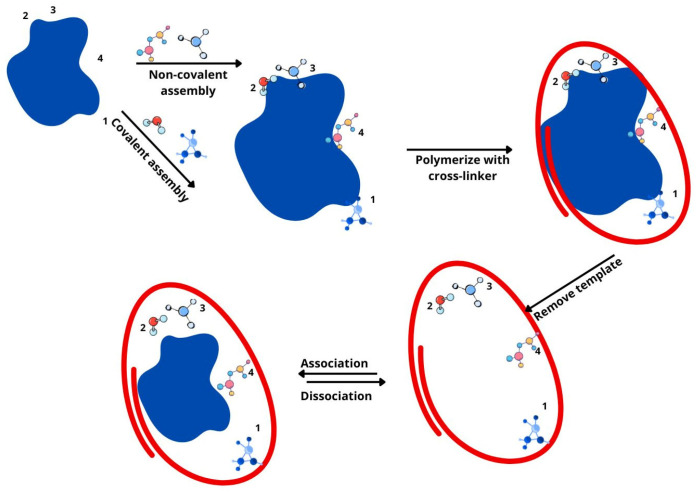
Schematic representation of MIP formation.

Molecular imprinting technology involves creating recognition sites within polymer networks that are specifically designed to bind template molecules. During polymerization, template molecules form covalent or non-covalent interactions with functional monomers, which cross-link to form copolymers. Once the template is removed, the resulting molecularly imprinted polymers possess cavities with precise recognition capabilities for the target molecule, enabling both physical and chemical binding [44] (Figure 2).

Molecular recognition is fundamental to numerous biological processes, particularly in biosystems. It involves dynamic receptor–ligand binding on the cell membrane, enabling cells to interact, transmit signals, and respond to external stimuli effectively [44]. The carcinoembryonic antigen (CEA) is a glycoprotein absent in healthy adults but expressed in various cancer tissues, including colorectal, breast, and pancreatic cancers. Researchers have developed sensors for detecting CEA using MIPs combined with electrosensors or optical devices, such as Raman spectroscopy with a pseudo-immune-sandwich assay. These methods show promise, but challenges remain, such as impaired performance in integrated systems and the need for simpler synthesis procedures for clinical use [45]. *H. pylori* is linked to various stomach-related diseases, including gastric cancer, with cytotoxin-associated gene A (CagA) being the primary virulence factor which enables its establishment in human gastric cells [46]. A novel MIP-based electrochemical sensor was developed for the ultrasensitive detection of the vacuolating cytotoxin A (VacA) toxin from *H. pylori*, a key factor in its pathogenesis. The sensor, featuring a SiO_2_ NP-decorated MIP on a screen-printed electrode, was created by polymerizing over the electrode using VacA as a template antigen, and it demonstrated high sensitivity and a low detection limit. The sensor effectively detected VacA with minimal interference from other substances within a linear range of 0.01–100 ng/mL [47]. A highly sensitive MIP-based electrochemical biosensor was developed for detecting CagA using a reduced graphene oxide and gold-coated, screen-printed electrode platform. The sensor exhibited high sensitivity, a low detection limit, and a linear range of 0.05–50 ng/mL, with minimal interference from other substances, demonstrating a strong binding affinity for the CagA antigen [46].

Celiac disease is a chronic immune-mediated condition of the small intestine triggered by dietary gluten in genetically predisposed individuals. Gluten ingestion in susceptible people causes inflammation in the upper small intestine, leading to mucosal damage which reduces nutrient absorption, including fat-soluble vitamins, iron, B_12_, and folic acid. At the same time, oats are typically non-immunogenic [48]. Diagnostics are pivotal in managing the increasing global prevalence of celiac disease and supporting the development of non-dietary therapies. Advances in understanding its immune pathogenesis and the role of serology have led to the partial acceptance of non-biopsy diagnosis in specific cases. This approach may expand further as methods for detecting gluten-specific celiac disease CD4^+^ T cells and the acute immune response to gluten ingestion become more accessible [49]. The evolving diagnostic landscape emphasizes current practices, limitations, the gluten challenge test, and the potential for diagnostics targeting the disease’s root cause—gluten-specific immunity [49]. In an era during which pharmaceuticals, including new drugs, repurposed treatments, and supplements, may complement the gluten-free diet in managing celiac disease, it is essential to evaluate the current diagnostic rigor, the limitations of dietary therapy, and the lack of objective markers for disease severity [50].

### 3.1. Biomarkers

Biomarkers are used in clinical practice and research to diagnose diseases, predict disease risks, monitor treatment success, identify patients who could benefit from specific medications, and anticipate potential side effects, offering a comprehensive view of cellular activities. In diagnostics, biomarkers form the foundation of in vitro tests, aiding in disease detection, monitoring progression, and predicting treatment responses, especially in precision medicine. Identifying and clinically validating biomarkers is essential for advancing diagnostics in various fields, including cancer, with liquid biopsies offering a promising, non-invasive alternative for diagnosis [51]. Identifying high-risk individuals or diagnosing early disease is a key aspect of primary prevention and a crucial strategy for effective treatment and improving overall survival [52]. Tumor biomarkers, produced by tumors or in response to tumors, play a critical role in cancer screening, early diagnosis, prognosis prediction, recurrence detection, and monitoring treatment efficacy. Advances in molecular biology technologies have significantly improved the discovery and detection of sensitive, specific biomarkers, contributing to personalized medicine and better cancer patient outcomes [33,53]. Biomarkers are essential tools for diagnosing GI disorders, serving as indicators of disease presence or severity. There are various diagnostic biomarkers, including serological, immunological, fecal, and genetic markers (such as non-coding RNAs), which have biological functions and diagnostic roles [54].

Biomarkers from fluidic consistency (Table 1) samples (blood, urine, stool, saliva) are most suited for MIP sensors, as such sensors are engineered to detect biomarkers’ molecules selectively. The biomarker’s molecule binds to the MIP (whereby chemical interactions (e.g., hydrogen bonding, van der Waals forces, electrostatic interactions) between the biomarker and the imprinted polymeric site occur), and a measurable signal is generated.

Although many potential biomarkers are still in the research phase and have not been validated for real-life clinical use in diagnosing specific GI diseases, their future prospects are promising. The variability in results from current studies highlights the need for large-scale, multicenter trials with standardized methods, as well as broader genomic coverage in diagnostic approaches [52]. Proteomic-based markers hold promise but are costly, limiting their routine use, while epigenetic markers, which can be detected in both bodily fluids and tissues, show great potential. However, due to their sensitivity to environmental factors and aging, they must be used cautiously, and a combination of genetic, epigenetic, and proteomic approaches is recommended for clinical practice in gastric tumor diagnosis and prognosis [113]. Advancements in molecular diagnostics, such as single-cell sequencing, are expected to improve accuracy, reduce costs, and increase the availability of biomarkers, making them a key component of personalized medicine in the near future [52].

MIP sensors mimic natural recognition mechanisms by creating synthetic binding sites within a polymer matrix that are complementary in shape, size, and chemical functionality [114] to the target biomarker; therefore, biomarkers can provide accurate, non-invasive diagnostics and real-time monitoring for conditions like colorectal cancer, IBD, and liver diseases. MIP-based sensors are particularly useful for detecting biomarkers due to their high selectivity, enabling precise binding to target molecules. This makes them invaluable in diagnostics. Several molecular imprinting techniques can be used to develop MIP-based sensors. For example, molecular imprinting (MI) develops selective recognition sites in polymers for specific target molecules [115]. It involves polymerizing a functional monomer around a template molecule, which is later removed to leave imprinted cavities. Electrochemical molecular imprinting combines MI with electrochemical techniques to detect target molecules via electrical signals [116]. Surface molecular imprinting (SMI) focuses on imprinting directly on a material’s surface, providing high sensitivity. Ryma et al. demonstrate that human monocyte-derived macrophages exhibit strong M2a-like pro-healing polarization when cultured on type I rat-tail collagen fibers, but not on collagen I films, suggesting the importance of 3D structural motifs in inducing macrophage polarization. The strategy of “melt electrofibrillation” was developed to create highly aligned nanofibrils of synthetic polymers resembling native collagen I, successfully triggering M2-like polarization in macrophages. These biomimetic fibrillar structures, particularly from poly(ε-caprolactone), induce macrophage elongation and polarization at a level comparable to interleukin-4 treatment [117]. Another study introduced a new ultrasensitive and universal “Raman indicator” sensing strategy for detecting protein biomarkers using a glass capillary-based molecularly imprinted SERS sensor. The sensor uses an inner SERS substrate layer for signal enhancement and an outer polydopamine imprinted layer for selective protein recognition, with imprinted cavities controlling the flow of a Raman indicator to reflect protein capture. This platform allows for the specific, reproducible detection of proteins, including trypsin enzyme, at low concentrations in biological samples without the need for sample preparation, offering a fast, general, and effective approach for point-of-care bioassays [118]. A new chiral discrimination strategy, the “inspector” recognition mechanism (IRM), is proposed using a chiral imprinted polydopamine (PDA) layer on a surface-enhanced Raman scattering (SERS) tag. The IRM works by detecting permeability changes in the imprinted PDA layer after chiral recognition, where the correct enantiomer fills the cavities, and the wrong enantiomer does not. An aminothiol inspector molecule is introduced to scrutinize the recognition status, decreasing the SERS signal only when it passes through vacant or non-specifically occupied cavities, ensuring the specific discrimination of chiral molecules regardless of their size, functional groups, or optical activities [119]. These strategies are widely used in biosensors to detect biomolecules, toxins, and pathogens.

To sum up, MIPs are synthesized by polymerizing functional monomers around a target template molecule, which is later removed, leaving specific recognition sites (Figure 2). These materials function by selectively binding to their target analytes through complementary interactions, mimicking natural receptors. MIPs offer advantages such as high selectivity, stability, and reusability, but they may have limitations in template removal, binding kinetics, and batch-to-batch reproducibility. Compared to classical methods like antibodies or enzymatic assays, MIPs provide a cost-effective and robust alternative, with recent advancements enhancing their sensitivity and applicability in various fields.

### 3.2. Molecularly Imprinted Polymers In Vivo

Recently, over the past quinquennial, MIPs have been increasingly integrated into complex in vivo studies, demonstrating their potential for biomedical applications. Researchers have investigated the biodistribution, clearance, cytotoxicity, and adjuvant properties of nanoMIPs in six-week-old pathogen-free Sprague Dawley rats following oral and intravenous administration [120]. The results showed that low-dose nanoMIPs were not rapidly sequestered by the reticuloendothelial system, persisted in tissues without major toxicity, crossed the blood–brain barrier, and were excreted through urine and feces [120]. Additionally, epidermal growth factor receptor (EGFR)-imprinted nanoMIPs exhibited weak adjuvant properties for ovalbumin, indicating both potential risks in therapeutic payload delivery and promising applications in immunotherapy [120]. MIPs have also been explored for targeted drug delivery in vivo. A magnetic MIP was synthesized and tested in a rheumatoid arthritis rat model. Using Fe_2_O_3_@mSi as the core for surface imprinting, dopamine as the monomer, and methotrexate loaded during polymerization, the MIP achieved a loading capacity of 201.165 ± 0.315 μmol/g. In a complete Freund adjuvant induced arthritis rat model, a 3D magnet-bearing construct facilitated targeted magnetic MIP delivery, significantly improving paw edema, paw diameter, gait score, and weight, as confirmed by histopathology [121]. These findings suggest that these MIPs effectively target inflammation sites and outperform free methotrexate in alleviating arthritic symptoms [121].

Further research has demonstrated the need for MIP-based innovations in oncology. Scientists developed a stealth radiation sensitizer using Au-embedded molecularly imprinted polymer nanogels (Au MIP-NGs) to enhance low-dose X-ray radiation therapy [122]. Surface plasmon resonance confirmed their strong affinity for human serum albumin, enabling the formation of an albumin-rich protein corona which granted stealth properties in vivo [122]. In a pancreatic cancer mouse model, Au MIP-NGs significantly improved radiation therapy efficacy, inhibiting tumor growth even at low X-ray doses (2 Gy) [122]. This approach underscores the potential of nanomaterial–protein interaction control for advancing cancer treatment, diagnostics, and theranostics [122]. Beyond oncology, MIPs are also being investigated for real-time in vivo monitoring applications. Researchers have developed fully reversible MIP sensors based on electrostatic repulsion, overcoming the slow-release kinetics of traditional MIPs [123]. By applying a small electrical potential, these sensors enable repeated analyte release and detection. A dopamine sensor with a 760 pM detection limit demonstrated high accuracy across 30 sensing-release cycles, successfully detecting <1 nM dopamine from PC-12 cells in vitro [123]. This innovation offers a reliable approach for continuous health monitoring and other charged-molecule sensing applications [123].

In gastrointestinal applications, a catheter-based MIP sensor was developed to measure histamine concentrations in the human duodenum, aiding in the diagnosis of gut disorders like IBS [124]. Utilizing impedance spectroscopy and MIPs, synthesized from acrylic acid monomers, the sensor maintained high specificity and stability in intestinal fluid. Validated in a simulated duodenal environment, the sensor demonstrated a detection range of 5–200 nM, corresponding to physiological and disease-associated histamine levels [124], making this platform have potential applications in cardiovascular, urological, gastrointestinal, and neurovascular diagnostics.

Despite promising results in animal models [125,126,127,128], future research should focus on innovative methods such as 3D organ models derived from stem cells. While large animal models, including primates, may still be necessary for final validation before clinical trials, efforts should prioritize reducing the use of animal testing where possible. The integration of MIPs into advanced in vitro models, organ-on-a-chip systems, and AI-driven simulations could further refine and accelerate their translation into clinical applications.

## 4. Perspectives of MIP-Based Sensors

The development of polymer science is particularly relevant, as polymers have long been used in bioscience and medical areas in various forms. Imprinting polymeric technologies at the molecular level paved the way for a new era of MIP. Originally designed to mimic the binding behavior of natural antibodies, MIPs have evolved to serve as synthetic receptors, replicating the function of a wide range of natural receptors in analytical applications. MIPs can be useful in diagnostics in several aspects: MIPs are used as recognition elements in biosensors, mimicking the specificity of antibodies for detecting biomarkers; MIPs can be functionalized with fluorescent or magnetic nanoparticles (NPs) for imaging-based diagnostics; finally, MIPs are employed as selective tools to isolate and concentrate specific analytes from complex biological samples such as blood, urine, or saliva. Electrodes, as more affordable and versatile components, can serve as effective sensors when coated with molecularly imprinted materials, enabling the selective detection of analytes through electrochemical signals. These sensors can be designed to target both large and small molecules, suitable for analytes with a wide range of molecular weights.

MIPs are highly suitable for gastroenterology detection because they selectively recognize and bind specific biomarkers, even in complex biological samples like gastric fluids. Their stability, cost-effectiveness, and adaptability to non-invasive sensor platforms make them ideal for advancing the early diagnosis and monitoring of gastrointestinal disorders. Therefore, it is important and beneficial to learn about the perspectives of MIPs. Natural receptors like antibodies and enzymes are highly specific but can be expensive, lack variability, and require careful storage. Synthetic receptors, such as aptamers, are also specific and sensitive but are costly to produce and may have limited stability in harsh conditions. In contrast, MIPs offer a distinct advantage in terms of cost-effectiveness, as they are synthesized through relatively simple, scalable processes using readily available materials. The diagnostic applicability of molecularly imprinted polymers (MIPs) can be compared to natural and synthetic receptors based on several key factors: specificity, sensitivity, stability, and cost-effectiveness. However, the specificity of MIPs can sometimes be lower than that of natural receptors, especially in complex biological samples.

MIP-based sensors are ideal for future personalized use due to their high selectivity for target biomarkers, even in complex samples, combined with their exceptional stability under varying conditions, reusability, and compatibility with diverse fluid types. Their versatility and scalability allow for miniaturization into portable or wearable devices, making them perfect for non-invasive, real-time, and continuous health diagnostics.

The rapid advancements in gastroenterology also highlight the growing demand for highly compatible implantable sensors and biosensors capable of detecting disease-specific biomarkers. Due to their superior biocompatibility and adaptability, polymer-based composites have demonstrated exceptional potential for applications such as scaffold development [129] and the incorporation of living cells [130]. These materials are especially relevant in addressing challenges in gastrointestinal disease diagnostics, where the sensitive and specific detection of biomarkers like C-reactive protein, fecal calprotectin, or mucosal antibodies is critical for early and accurate disease detection [131,132]. By improving the biocompatibility of conducting polymer-based structures, researchers have paved the way for safer and more effective biomedical tools. For instance, these materials have shown promise in gastrointestinal therapeutic applications, such as localized drug delivery systems and bioresorbable sensors for monitoring intestinal health [133,134]. Therefore, by mimicking biological receptors, MIP sensors can selectively bind to biomarkers associated with gastrointestinal diseases, including specific proteins, metabolites, and microbial markers. Their robust and customizable design allows for enhanced sensitivity and specificity, making them ideal for real-time monitoring in complex biological environments such as the gut [135].

Composite materials, especially hydrogels derived from conducting polymers, have emerged as highly biocompatible options due to their significant water content [136]. Their biocompatibility can be significantly enhanced by incorporating compatible materials for synthesis and modification as well [137]. The exceptional biocompatibility of these hydrogel-based composites paves the way for their application in attachable [138], wearable [139], and other advanced biosensors [140].

The potential of conducting polymers in developing advanced biomedical devices particularly in the area of gastrointestinal diseases is growing. Research has shown that conducting polymers, such as polypyrrole, demonstrate significant compatibility with various entrapped proteins [141,142]. For instance, polypyrrole has been used to immobilize glucose oxidase, maintaining the enzyme’s activity and facilitating electron transfer in glucose biosensors [143]. Monitoring blood glucose levels is crucial in managing the gastrointestinal complications associated with diabetes, such as gastroparesis. Gastroparesis, characterized by delayed gastric emptying, can lead to unpredictable glucose absorption, resulting in erratic blood sugar levels [144]. Additionally, polypyrrole’s lack of negative effects on hematological parameters reinforces its suitability for biomedical applications. Research involving polypyrrole NPs has shown no significant cytotoxicity, indicating their potential for safe use in medical devices. Other conducting polymers, such as polyaniline and polythiophene, as their derivatives, are also known for their compatibility in biomedical applications. These materials have been explored for their biocompatibility and potential use in various tissue engineering and biosensing applications [145].

Despite the critical need for biocompatibility in implantable devices, most studies in this field have focused on evaluating the compatibility of conducting polymers with basic biological molecules such as enzymes and DNA [146,147]. However, such studies often fall short of assessing the intricate biocompatibility essential for clinical applications. Advanced biocompatibility testing involving cell lines or animal models is necessary to address this limitation comprehensively. The adaptability of MIPs allows them to be used in conjunction with cell line and animal model experiments. For example, MIPs could monitor localized cellular responses or inflammatory markers, bridging the gap between in vitro and in vivo biocompatibility evaluations. MIPs can be fabricated using various functional monomers and cross-linkers, which enable the design of materials with specific mechanical and chemical properties [148]. This versatility ensures that the sensors meet the stringent requirements of implantable medical devices. MIPs can be engineered to interact with specific molecules in a controlled manner. By incorporating biocompatible polymers during the imprinting process, MIPs can enhance compatibility with living tissues and reduce adverse reactions in implantable devices. Despite the opportunity to improve the binding affinity in vitro, the application of MIPs in vivo remains challenging. Several strategies can be employed to optimize MIPs, such as optimizing the functional monomers, cross-linker modification, and solvent selection during polymerization. In addition, post-imprinting treatments like solvent washing, heat activation, or the incorporation of nanomaterials can increase surface area and enhance binding capacity. However, biological matrices, such as blood and tissues, contain interfering molecules that lead to non-specific binding, reducing the MIP’s recognition capability. Due to degradation by enzymes or environmental factors, the stability of MIPs can be decreased in vivo. The size and accessibility of the target molecules limit MIP effectiveness. Large MIPs may not penetrate tissues, while small MIPs may lack sufficient binding sites.

It is important to emphasize that the toxicity, biocompatibility, and biodegradability of MIPs for in vivo applications should be enhanced. Using biocompatible monomers and polymers might reduce immune responses and improve MIP safety [149]. Incorporating biodegradable materials such as poly(lactic acid) or poly(ε-caprolactone) into the MIP structure allows for controlled degradation over time, reducing long-term accumulation in the body [150,151]. To ensure safe in vivo applications, in vitro testing should be conducted to assess the long-term effects and clearance of MIPs from the body.

In addition, significant advancements in MIP-based diagnostic tools have not yet been tested in clinical trials. The main reasons might be associated with biocompatibility and safety concerns. Understanding of in vivo interactions with nanoMIPs is still limited, especially regarding the impact of binding cavities on internalization, biodistribution, and clearance. This knowledge gap highlights the need for further research into the behaviors of nanoMIPs once they enter the bloodstream and interact with blood components, tissues, and cellular microenvironments [120]. In order to translate created medical devices to clinics, comprehensive safety and efficacy examination is needed, including pre-clinical and clinical trials. These regulatory frameworks could be challenging to undergo and might delay the approval and adoption of novel technologies [152]. Developing high-sensitivity and -selectivity MIPs in complex biological matrices, such as gastrointestinal fluids, remains challenging. The presence of various interfering substances can affect the binding affinity and specificity of MIPs, complicating their performance in real-world clinical settings [153].

## 5. Conclusions and Future Perspectives

In conclusion, molecularly imprinted polymers represent a promising advancement in the field of gastrointestinal medical diagnostics. Their ability to selectively bind target molecules makes them valuable for detecting biomarkers associated with gastrointestinal disorders. Customizing MIPs to recognize specific analytes enhances their sensitivity and specificity, paving the way for more accurate diagnostic tools. Moreover, the stability and durability of these polymers facilitate their use in harsh gastrointestinal environments, ensuring reliable performance. As research progresses, integrating MIPs with advanced detection technologies based on the application of sensors will likely amplify their diagnostic capabilities. This synergy of electronics and MIPs could lead to rapid, point-of-care testing that improves medical treatment outcomes through timely interventions. Additionally, MIPs can be designed to target a wide range of compounds, ranging from proteins to small metabolites, broadening their applicability in gastrointestinal diagnostics. The implementation of MIPs in clinical settings has the potential to revolutionize gastrointestinal diagnostics, enhancing early detection and personalized treatment strategies. However, some challenges in this area still remain, including the need for regulatory approval and large-scale production, but ongoing innovations are addressing these hurdles. As technological progress moves forward, continued research and collaboration will be crucial in unlocking the full potential of molecularly imprinted polymers in healthcare.

## Figures and Tables

**Table 1:** Fluidic samples for biomarker detection.

Fluid	Biomolecules	Biomarkers	Ref.
Blood-Based Biomarkers	Proteins	Carcinoembryonic Antigen (CEA): Commonly elevated in colorectal cancer. CA 19-9: Associated with pancreatic and biliary cancers. Alpha-Fetoprotein (AFP): Used in hepatocellular carcinoma diagnosis. C-Reactive Protein (CRP): Indicates inflammation in conditions like inflammatory bowel disease (IBD). Serum Albumin: Lower levels are often linked to liver disease.	[17,55,56,57,58,59,60,61,62,63,64,65]
	Enzymes	Amylase and Lipase: Indicators of pancreatic inflammation (e.g., pancreatitis). Aspartate Transaminase (AST) and Alanine Transaminase (ALT): Reflect liver function.	[66,67]
	Cytokines	Tumor Necrosis Factor-Alpha (TNF-α): Elevated in IBD and Crohn’s disease. Interleukins (e.g., IL-6, IL-8): Markers of inflammation and cancer progression.	[68,69,70,71,72,73]
	Nucleic Acids	Circulating Tumor DNA (ctDNA): Useful for detecting genetic mutations in GI cancers. MicroRNAs (e.g., miR-21): Associated with gastric and colorectal cancers.	[74,75,76,77,78,79,80]
	Metabolites	Bilirubin: Indicates liver function or obstruction in bile ducts. Lactate: Can reflect hypoxia or tumor metabolism in cancers.	[81,82,83,84]
Saliva-Based Biomarkers	Proteins	Cytokines (e.g., IL-8): Indicators of oral and esophageal cancers or systemic inflammation. Amylase: Reflects salivary gland or pancreatic function.	[85,86,87,88,89]
	DNA/RNA	MicroRNAs (e.g., miR-21): Associated with GI cancer detection.	[90,91]
Stool-Based Biomarkers	Proteins	Fecal Calprotectin: A marker for IBD and colorectal cancer. Fecal Immunochemical Test (FIT): Detects occult blood in stool, used in colorectal cancer screening.	[92,93,94,95,96]
	DNA/RNA	Methylated DNA (e.g., SEPT9): Found in stool for colorectal cancer screening. Microbial DNA (e.g., alterations in gut microbiome composition): Associated with various GI diseases.	[22,97,98,99,100,101]
Urine-Based Biomarkers	Proteins	Urinary Peptides (e.g., MMP-9): Linked to gastric cancer. Nitrites: May indicate infection (e.g., Helicobacter *pylori*).	[102,103,104,105]
	Metabolites	Volatile Organic Compounds (VOCs): Associated with colorectal cancer and GI inflammation.	[106,107,108]
	Genetic Material	Urinary MicroRNAs (e.g., miR-92a): Indicators of colorectal cancer.	[109,110,111,112]

## Data Availability

Data sharing does not apply to this article as no datasets were generated.

## Abbreviations

The following abbreviations are used in this manuscript:

AFP	Alpha-Fetoprotein
ALT	Alanine Transaminase
ANCAs	Anti-neutrophil Cytoplasmic Antibodies
ASCAs	Antifungal Brewer Yeast Antibodies
AST	Aspartate Transaminase
CEA	Carcinoembryonic Antigen
CD	Crohn’s Disease
CRP	C-Reactive Protein
ctDNA	Circulating Tumor DNA
ESR	Erythrocyte Sedimentation Rate
FIT	Fecal Immunochemical Test
GC	Gastric Cancer
GI	Gastrointestinal
IBD	Inflammatory Bowel Disease
IFN-γ	Interferon-Gamma
IL	Interleukin
LF	Lactoferrin
MIP	Molecularly Imprinted Polymer
miR	MicroRNA
NP	Nanoparticle
Omp-C	Escherichia coli Outer Membrane Porin C
PKM2	Pyruvate Kinase M2
SARS-CoV-2	Severe Acute Respiratory Syndrome Coronavirus 2
TNF-α	Tumor Necrosis Factor-Alpha
Tregs	Regulatory T Cells
UC	Ulcerative Colitis
VOCs	Volatile Organic Compounds
